# Early Occupational Therapy Intervention Programme and Coping Strategies and Burden in Caregivers of People with Sub-Acute Stroke: A Randomised Controlled Trial

**DOI:** 10.3390/jpm14080821

**Published:** 2024-08-01

**Authors:** Patricia García-Pérez, María Carmen Rodríguez-Martínez, Alejandro Gallardo-Tur, Encarnación Blanco-Reina, Carlos de la Cruz-Cosme, José Pablo Lara

**Affiliations:** 1 Department of Physiology, Faculty of Medicine, University of Malaga, 29016 Málaga, Spain; 0619043053@uma.es (P.G.-P.); jplara@uma.es (J.P.L.); 2 Occupational Therapy Department, Hospital Civil, Servicio Andaluz de Salud (SAS), 29009 Málaga, Spain; 3 Brain Health Unit, Centro de Investigaciones Médico-Sanitarias (CIMES), 29010 Málaga, Spain; eblanco@uma.es; 4 Department of Physiotherapy, Faculty of Health Sciences, University of Malaga, 29071 Málaga, Spain; 5 Biomedical Research Institute of Malaga-Nanomedicine Platform (IBIMA-BIONAND Platform), 29590 Málaga, Spain; carlos.cruz.sspa@juntadeandalucia.es; 6 Neurology Department, Virgen de la Victoria University Hospital, 29010 Málaga, Spain; alejandro.gallardo.sspa@juntadeandalucia.es; 7 Department of Pharmacology and Therapeutics, Faculty of Medicine, University of Malaga, 29016 Málaga, Spain; 8 Department of Medicine and Dermatology, Faculty of Medicine, University of Malaga, 29016 Málaga, Spain

**Keywords:** occupational therapy, caregiver, quality of life, rehabilitation, stroke

## Abstract

Caregivers of people who have suffered a stroke experience a great burden and may use disengagement coping strategies. We studied the influence of an early occupational therapy intervention programme in the process of hospital-to-home discharge after stroke (EOTIPS) in a Spanish cohort that improved patients’ quality of life and caregivers’ burden and coping strategies. EOTIPS was delivered by a single occupational therapist. We conducted a prospective randomised controlled trial that included 60 adults who suffered a stroke, of which 91.6% had a caregiver who agreed to be involved in their care (*n* = 55). Evaluations assessed the caregivers’ burden and coping strategies within two weeks post-stroke and after a three-month follow-up. Statistical analyses included intent-to-treat analysis (considering dropouts as failures) and efficacy analysis, considering only end-of-treatment participants. The caregivers in the intervention group showed a significantly better evolution in the main outcome measure of burden (*p* = 0.019), as well as in the coping strategies of social support (*p* = 0.037) and social withdrawal (*p* ≤ 0.001), compared with the control group. EOTIPS was effective in improving the caregivers’ burden and two coping strategies, and it could be considered as an applicable tool that can minimise the risk of suffering burden.

## 1. Introduction

Caregivers of people who have suffered a stroke usually experience a great burden and may use disengagement coping strategies such as avoidance or distancing [1]. This negative assessment of the situation is key when experiencing a burden [2]. It is essential for caregivers to improve coping skills and social support to avoid blame and denial and seek instrumental and emotional support [3]. Interventions should enhance caregivers’ ability to cope with stress and improve their skills to reduce patients’ physical dependence [4].

A descriptive study highlighted the need to develop appropriate coping strategies in caregivers to achieve a reduction in their state of anxiety regarding the problem [5]. Guiding the caregivers of stroke patients towards a positive coping style reduces the stress responses caused by caregiving [6]. These types of strategies predict a better health-related quality of life [5]. Professionals must attend to the coping abilities of caregivers, their emotional state, and the level of dependency of patients since they are vital and modifiable factors that affect the burden on caregivers [2,7].

Caring for patients with stroke places a great burden on caregivers, which manifests itself in depression and anxiety [8]. The burden has been defined as the level of multifaceted strain perceived by a caregiver from caring for a family member or loved one over time [9]. Specifically, in our study, caregiver burden focused on the burden related to the consequences of a stroke and the status of patients’ conditions as influences on caregivers’ physical health and psychological well-being [1,10]. When it comes to caregivers of people with stroke, they seem to present a greater burden and lower resourcefulness, along with depression and anxiety, compared with caregivers of people with other diseases [11]. Stress is present in caregivers of stroke victims since they need physical, psychosocial, and educational support to be able to help users and thus mitigate the burden experienced [9]. Greater caregiver burden is associated with greater emotional distress and lower functionality in stroke patients [4,12]. Programmes should be designed to promote the skills of caregivers and thus reduce their level of burden [1], as higher caregiver burden is associated with lower family resilience [13]. The findings suggest that consistent interventions, such as emotional support or counselling on stress-relief strategies for caregivers of stroke survivors, may reduce caregiver burden [12]. Targeted interventions aimed at improving family resilience and coping mechanisms are crucial to optimising the well-being of both caregivers and patients [14]. Homecare provided to stroke patients and education on disability and economics for their caregivers reduce the burden of care and increase the quality of life for both parties [15,16]. Therefore, psychological counselling and social support should be provided to caregivers to reduce their physical and mental burden [17,18].

As part of the Early Occupational Therapy Intervention Post-Stroke (EOTIPS) programme [19,20], the influence it had on patients’ recovery was studied, showing, in the final results, that it improved patients’ quality of life, as well as patients’ independence, perceptual–cognitive skills, and symptoms of depression, compared with the control group (article pending for acceptance). Most of the similar research that has been previously studied has incorporated the caregiver as a potentiality for rehabilitation, increasing the patient’s adherence to treatment and leading to a reduction in the cost of treatment and rehabilitation [19]. Nevertheless, some studies have been unable to prove that caregiver education on its own can significantly improve patients’ functional abilities [21].

The general objective of the current study was to explore the influence of EOTIPS in a Spanish cohort on improving patients’ quality of life and caregivers’ burden and coping strategies. We hypothesised that caregivers in the intervention group would present a significantly better evolution in the main outcome measure of burden, as well as in some of the coping strategies, and that this better evolution could also be related to the patients’ symptoms of depression and anxiety.

## 2. Materials and Methods

### 2.1. Study Design

This research was conducted in parallel to EOTIPS, which was a prospective, longitudinal, randomised, and controlled clinical trial, the protocol of which was previously defined and published in [20]. The caregivers of the experimental group participants who were included in the EOTIPS programme (*n* = 28) were compared with the caregivers of the control group participants (*n* = 27). The Malaga Research Ethics Committee (CEI) approved the current research on 25 February 2021. This study was registered at ClinicalTrials.gov with the identifier NCT04835363. The results are reported in accordance with the CONSORT guidelines (see Table S1: CONSORT 2010 checklist) [22].

### 2.2. Participants and Recruitment Procedures

Recruitment started on 25 May 2021 and finished on 30 November 2022. Patients and caregivers assigned to the experimental group were included in EOTIPS, a programme in which they received early occupational therapy intervention, and were compared with a control group. Both groups received the usual care and rehabilitation provided by other health professionals. Participants were randomly assigned to each group according to a computer’s pre-established designation in blocks of six patients, developed by the principal investigator of the study with the online tool Sealed Envelope, v1.23.0 [23]. Masked medical staff of the neurology department were responsible for selecting and referring suitable patients to the occupational therapist to participate in the study. Every participant was given a number to codify the evaluation envelopes, which matched their data in the database. Blinded evaluators carried out the assessments, and patients and caregivers were not aware of their group allocation. Only those who signed a written informed consent form were included in this study.

In the current analysis, we examine the influence that inclusion in the EOTIPS study had on the caregiver burden and coping strategies, comparing the experimental group (caregivers of patients who participated in EOTIPS but also received conventional care and support) with the control group (caregivers of patients who received conventional care and support). Therefore, the only inclusion criteria were as follows: (1) participants of EOTIPS—therefore, patients with a diagnostic confirmation of stroke, being >18 years old, with >2 to <26 points on the National Institute of Health (NIHSS) scale, who present some motor or cognition deficit that made it difficult to carry out activities of daily living (ADL) and who are going home on discharge; and (2) caregiver availability (relative or friend) who is happy to be deeply involved in their care and participate in the research. Exclusion criteria were as follows: (1) life expectancy <1 year, (2) participants who had suffered a previous stroke or other major medical illnesses that could alter cognitive function, (3) absence of a caregiver, and (4) people who do not understand Spanish or English.

Inclusion in the study occurred prior to hospital discharge. The EOTIPS intervention started as soon as patients were medically fit for it, and the initial assessment was completed, in all cases, within two weeks after the stroke. During data collection, the anonymity of patients and caregivers was guaranteed. Participants were informed of the intervention and were asked to sign a written informed consent, since the participation was voluntary, allowing the user to leave whenever they wished. The principles of the Declaration of Helsinki were followed [24].

### 2.3. Interventions

The current research aimed to reduce caregivers’ burden, which occurred in parallel to EOTIPS research, whose main objective was to improve patients’ quality of life by facilitating the transition from hospital to home. The intervention group participated in EOTIPS, which was delivered by a single expert occupational therapist with advanced training in neurology. It took place both in the hospital and in the patient’s home. Firstly, the occupational therapist, together with the patient and their caregiver, explored limitations and potentialities to set individual goals for the intervention period. EOTIPS included an initial evaluation, a first session in the hospital before discharge, a post-discharge home visit, a phone follow-up, a home visit one month later, and a final evaluation three months after discharge. Therefore, the occupational therapist communicated frequently with patients and caregivers via home visits and phone calls to offer information and support during the transition from hospital to home and the start of the stroke sub-acute period. The overall aim was to educate patients and caregivers in basic neurorehabilitation and to motivate caregivers to lead rehabilitation at home following OT instructions. The OT provided information regarding postural care and transfers, cognitive/motor/perceptual/sensory stimulation, basic neurorehabilitation exercises, the importance of patient involvement in significant activities and basic/instrumental (ADL), and recommendations to perform ADL safely at home. This intervention was carried out in parallel to the standard of care provided by the healthcare system when the trial took place. The standard care provided at the time of the research by the healthcare system included inpatient physiotherapy (one 30 min session a day, five days a week), while OT was not provided for inpatients. The rehabilitation doctor evaluated patients just before discharge and could refer them to physiotherapy and OT. Nevertheless, the OT waiting list was long, and participants did not get to start outpatient OT rehab before the end of the research follow-up.

### 2.4. Outcomes

Evaluations assessed the caregiver’s coping strategies (Coping Strategies Inventory [CSI]) and caregiver’s burden (Caregiver Burden Scale [CBS]) of both groups. Evaluations were completed within two weeks post-stroke and after three months follow-up. Statistical analysis included intent-to-treat analysis, considering all caregivers (dropouts as failures), and efficacy analysis, considering only end-of-treatment participants’ caregivers. Evaluators were OT practitioners previously trained to carry out the assessments and who were blinded to group assignment. Initial assessments were performed in the hospital, while final assessments took place at patients’ homes three months after discharge. All measures used in the research were standardised, validated, and adapted to the Spanish language. Caregivers’ burden and coping strategies were assessed with the following evaluations:

Caregiver Burden Scale (CBS) is a 22-item scale that assesses subjectively experienced burden by caregivers. Originally designed and tested in 1980 with 29 items, it was reduced to 22 questions [25,26]. The caregiver is asked to tick one of the four boxes (not at all, seldom, sometimes, often) and score 1 to 4 for each question. The instrument comprises five factors: general strain, isolation, disappointment, emotional involvement, and environment. Data can be expressed as mean values for the total scale as well as mean scores for the five different dimensions. The total score range is 0 to 88, where the higher the score, the more severe the burden is [26]:

0–21: no to mild burden.
21–40: mild to moderate burden.
41–60: moderate to severe burden.
≥61: severe burden.

Coping Strategies Inventory (CSI) was initially a 72-item scale generated by Tobin et al. in 1989 and later adapted by Cano et al. in 2007 into 40 items [27,28]. This scale measures eight subscales with primary strategies: problem-solving, self-criticism, emotional expression, illusions, social support, cognitive restructuring, problem avoidance, and social withdrawal. Subjects are asked to generate a description of a specific stressful event and then to indicate the extent to which they used specific coping responses, using a 5-item Likert format [28,29]. Individual coping strategies are broadly classified into two basic approaches to managing stressful situations: coping activities that engage the individual in the stressful situation and coping activities that disengage the individual from it [30].

A semi-structured survey designed for this study was completed at the end of the research, which included a Likert scale and a dichotomous scale with the aim of obtaining qualitative data regarding patients’ and caregivers’ satisfaction. Within the same project, the patients’ variables of quality of life, symptoms of depression, and anxiety were assessed with the following standardised evaluations: Spanish Aphasia and Quality of Life Scale-39 (SAQOL-39), Beck Depression Inventory-II (BDI-II), and Hamilton Anxiety Scale (HAM-A).

### 2.5. Statistical Analysis

The patients’ sample size “*n*” was calculated to represent the total population of patients who suffered a stroke and were admitted to a second-level hospital in Málaga, Spain, and who presented some post-stroke sequelae that made it difficult to carry out ADL. It was based on EOTIPS primary outcome SAQOL-39 [31] with the sample size determination formula for two independent groups, assuming a mean of 2.50, standard deviation of 0.75, significance level of 5%, a power of 80%, and a critical difference of 0.54, obtaining a sample of 30 participants per group and a total of 60 participants. Therefore, two groups were formed: an intervention group of 30 patients who received EOTIPS, of whom 29 had a caregiver who participated in the research, and a control group of 30 patients who received conventional rehabilitation and care, of whom 28 had a caregiver. Therefore, a total *n* of 57 patients with their caregivers were evaluated and analysed to answer the current research question.

To compare changes between the control and the experimental group, a repeated measures ANOVA was performed considering a within-subject factor (Time) and a between-subjects factor (Group). Where ANOVA factors were significant, post hoc pairwise comparisons (Tukey test) were performed. For all tests, a *p*-value of <0.05 was considered statistically significant. The effect sizes for the significant findings (Cohen’s d) were added to the results table. Simple linear regression analyses were performed using CBS results as the dependent variable. For the statistical analysis of qualitative variables within the semi-structured survey, Pearson’s Chi-squared tests were used. The software used for data analysis was JAMOVI, version 2.3.26 [32].

## 3. Results

### 3.1. Recruitment and Participant Characteristics

We conducted a prospective, randomised, controlled clinical trial that included 60 adults who had suffered a stroke and were discharged home (see Table 1). Although our study aimed to incorporate the caregiver in the rehabilitation, five of the included patients did not have a relative or close friend willing to be deeply involved in their care. Therefore, 91.6% had a caregiver who agreed to take on this role (*n* = 55), in most cases the patient’s companion, as 61.6% were married or living with their partner. Furthermore, a significant percentage of caregivers were sons or daughters, although there were also private caregivers and other relatives (see Table 2).

**Figure 1:**
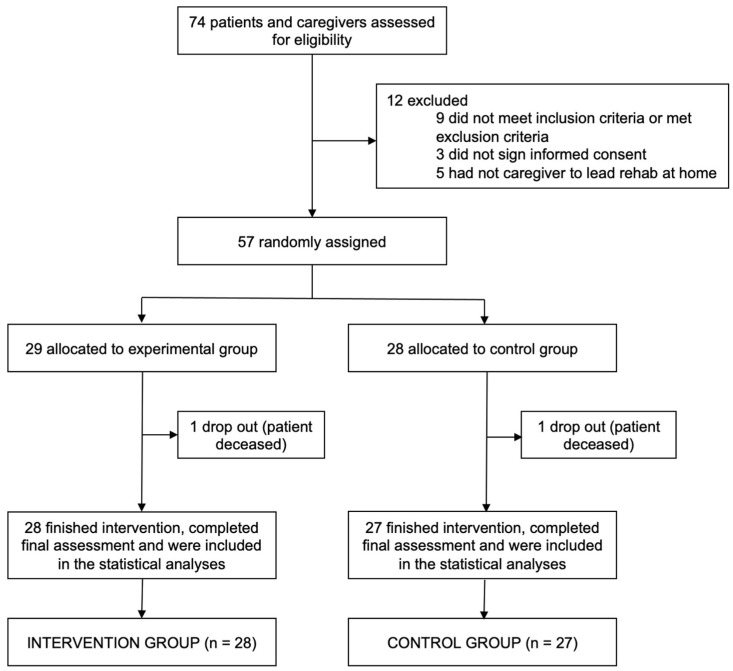
Trial profile.

Out of the 84 initially screened potential participants, 65 were eligible to participate in the trial. However, 3 patients declined to sign the written informed consent, and out of the 60 patients who completed the study, only 57 had a relative or friend as a caregiver and agreed to cooperate with the research. Finally, two participants died before completing the study. An overview of the flow of participants through the study is provided in Figure 1.

Recruitment ended after 18 months, once the sample size was reached, and all patients and caregivers entered the study when patients were in the acute phase of their first stroke.

### 3.2. Outcome Analysis

A repeated measures ANOVA was conducted to examine the change of the variables from baseline to final evaluation on the evaluation scores, assessing whether there was a significant interaction between the time of evaluation (within-subject factor) and group (between-subjects factor) (see Table 3). The means and standard deviations in the initial and final evaluations for each group were included, as well as the analysis results to verify significant differences between both groups, including *p*-values, F-values, degrees of freedom (df), and effect sizes (*d*) for the significant findings.

The repeated measures ANOVA results indicated that participants in the intervention group showed significantly better improvement in the main outcome measure of burden, CBS (F = 6.04, *p* = 0.019), as well as for two out of the eight primary coping strategies assessed by CSI: social support (F = 4.68, *p* = 0.037) and social withdrawal (F = 13.88, *p* ≤ 0.001), compared to the control group. Neither of the two approaches of the CSI nor the other six coping strategies assessed by CSI was statistically significant when comparing the control and experimental groups, although both groups showed a significant increase in their engagement strategies (F = 11.60, df = 1.36, *p* = 0.002). Post hoc pairwise comparisons revealed that evaluation scores were not significantly different between the control and experimental groups for the baseline evaluation (T1) but were for the final evaluation (T2) of one of the primary coping strategies assessed by CSI, social withdrawal (T1 *p* = 0.996; T2 *p* ≤ 0.001). The effect sizes for the significant findings ranged from medium to large effects.

Simple linear regression analyses were conducted with the CBS results as the dependent variable, revealing significant results with the covariates SAQOL-39 (*p* ≤ 0.001, R^2^ = 0.291), HAM-A (*p* = 0.004, R^2^ = 0.191), and BDI-II (*p* = 0.002, R^2^ = 0.230). Consequently, there is an important relation between the patients’ quality of life, symptoms of depression and anxiety, and the level of burden experienced by caregivers.

The semi-structured survey completed at the end of the research showed that most caregivers (78.6% of experimental group caregivers versus 59.3% of control group caregivers) reported that care difficulties had reduced over time, although differences between groups were not significant (see Table 4). Additionally, the survey indicated that the control group consulted stroke-related issues with their GP or neurologist more than twice as often as the experimental group (35.7% of the experimental group versus 74.1% of the control group patients and caregivers), and this difference was statistically significant (*p* = 0.004). The Likert scale included in the semi-structured survey showed statistical differences for two variables, opinions regarding rehabilitation (*p* = 0.011) and home care (*p* < 0.001) received by public healthcare, with the experimental group showing greater satisfaction than the control group (see Table 5).

Out of the 28 caregivers and patients who participated in the experimental group, 82.1% rated the care received as 5 out of 5, and the remaining 17.9% rated the care as 4 out of 5 (see Table 5). Finally, it is worth mentioning that no harm or unintended negative effects were generated or reported during the trial.

### 3.3. Study Variables

The values of the burden variable examined with CBS varied from baseline to final evaluation for the two groups. At baseline, 75.61% of caregivers experienced no burden or mild burden, 21.05% experienced mild-moderate burden, and only 3.5% experienced moderate–severe burden, with none reporting severe levels of burden. The average burden score was ±13.65. At the time of the final evaluation, 77.19% of caregivers experienced no burden or mild burden, and 22.8% experienced mild–moderate burden, with no caregiver reporting moderate–severe or severe levels of burden. This indicates a lower frequency of burden experiences compared to the initial evaluation. The average burden score in the final evaluation was ±0.66.

According to CSI results, it was found that among the most commonly used strategies at the baseline evaluation, just after the stroke occurred, were wishful thinking (84.21%) and problem-solving (71.05%), while the least used were problem avoidance (15.79%) and self-criticism (18.42%). Similarly, the most commonly used strategies at the time of the final evaluation, three months after discharge, were the same strategies but with varying frequencies: wishful thinking (86.84%) and problem-solving (83.95%), while the least used were problem avoidance (21.05%) and self-criticism (26.32%). Therefore, while engagement coping mechanisms related to problem-solving improved over time, the frequency of disengagement strategies, such as self-criticism, also increased.

## 4. Discussion

The present study examines the interaction between EOTIPS, caregiver burden, and coping strategies. The experimental group reported less burden than the control group, which correlates with better quality of life for the patients. The engagement coping strategy of social support also showed statistically significant differences between groups, as it increased more for the experimental group, indicating they reported better social support. Similarly, the disengagement coping strategy of social withdrawal showed statistically significant differences between groups; it increased in the control group but decreased, albeit minimally, in the experimental group. A statistically significant relationship was also revealed between patients’ quality of life, their symptoms of depression and anxiety, and the level of caregivers’ burden. Specifically, better patient quality of life correlated with lower caregiver burden, while higher patient levels of depression and anxiety were associated with higher caregiver burden. The quality of the relationship between the family member and the stroke survivor also influences long-term functional outcomes [7]. This is because caregivers play a crucial role in supporting patients who have suffered a stroke and often experience mental disorders and a high care burden, negatively affecting their quality of life [33]. Similarly, a longitudinal study shows how the quality of life of a person with stroke affects their caregiver’s burden, anxiety and depression [34]. This is supported by the results of a cross-sectional pilot study that found that physically and emotionally affected caregivers were associated with lower patient quality of life [7,35]. Therefore, caregivers’ well-being can either improve or hinder the patient’s recovery [36]. Stroke also impacts the socioeconomic status of caregivers due to patient dependency and its consequent influence on the caregiver’s health and finances, which are risk factors for a low quality of life [16,37,38]. A randomised controlled trial shows that caregivers’ education reduces their burden and improves psychological outcomes [39]. Furthermore, educating caregivers on a flexible coping style leads to a significant increase in quality of life [40]. The level of stress experienced by caregivers is also high upon discharge, so caregivers must be advised and trained to meet the needs and uncertainties of caring for a stroke patient [37]. This supports the implementation of the EOTIPS intervention during the discharge process. The occupational therapist trained the caregiver on postural care and transfers, basic neurorehabilitation exercises, and useful equipment and provided recommendations for safely performing ADLs at home. Caregivers were encouraged to discuss the patients’ struggles at home and their difficulties with the assigned occupational therapist, who offered recommendations to support them through the discharge process after stroke.

### 4.1. Burden

There is little research on evidence-based interventions aimed at reducing the risk of caregiver burden [10]. Currently, most rehabilitation programmes focus primarily on stroke patients rather than caregivers, and the educational needs of caregivers often receive insufficient attention [41]. The clinical trial by Loh et al. [42] highlights the reduction in family tension in caregivers who received a social support programme, which could also lead to improved patient quality of life, as suggested by the correlations observed in the current study. Similarly, a cross-sectional study collected anxiety, depression, and burden scores of caregivers using the HAM-D, HAM-A, and CBS scales, demonstrating a relationship between caregiver burden and depression and anxiety [17]. An RCT aimed to explore the association between caregiver burden and psychological distress, the amount of care provided, and its impact on the patient’s physical deterioration [43]. Although it showed a statistically significant improvement in motor evaluations, CBS did not reveal significant differences between the groups [43]. In the current study, both groups showed great improvements in CBS scores, indicating the substantial impact of stroke on caregivers’ lives. The results suggest that there was a significant difference between groups, which may be attributed to the treatment applied to the experimental group (see Table 3). Previously published data emphasise that caregivers with a higher burden of care may use more negative coping strategies, such as escape–avoidance and distancing. Effective coping skills can reduce personal burdens and improve caregivers’ physical health and psychological well-being. Therefore, to encourage caregivers to use engagement coping skills, appropriate programmes should be designed and implemented to support them [1]. It is crucial to understand how support given to both patients and caregivers can influence the caregiver burden and coping strategies, as well as improve patient quality of life, which were the main objectives of the current research.

### 4.2. Coping Strategies

Regarding the coping strategies, the current research shows little difference between the groups, as no statistically significant differences were found for the two main approaches: engagement strategies and disengagement strategies (see Table 3). On the other hand, two out of the eight subscales showed significant differences between the control and the experimental group, social support and social withdrawal, with the latter being the only subscale that showed a significant difference in the post hoc comparison. As seen in Table 3, social withdrawal increased in the control group, which suggests that without adequate support, caregivers might avoid social interactions, potentially increasing their levels of burden. Previously published studies have shown the advantages of psychoeducational programmes for caregivers of stroke patients in reducing caregiver burden and improving coping strategies, which can enhance family interaction, problem resolution, and social support, consequently reducing the caregiver’s burden in the intervention group [44,45].

### 4.3. Association between the Caregiver Scores and Patient Quality of Life

Association results indicate a relationship between the psychological well-being of family caregivers and improved outcomes for stroke survivors [46]. In the current study, simple linear regression analyses were conducted using the CBS results as the dependent variable, revealing a significant result with SAQOL-39 results (*p* < 0.001, R^2^ = 0.291). It is important for both the patient and the caregiver to have a strong support network, which can alleviate the burden on the primary caregiver and improve patient outcomes. Therefore, the importance of supporting and evaluating patients together with their caregivers is emphasised to prevent the emergence of negative affective states [8]. Moreover, the situation of caregivers varies depending on the patient’s level of dependency, so the needs of caregivers cannot be generalised. It is therefore necessary to provide individual support for those who care for people with greater dependency [47].

### 4.4. Qualitative Section of EOTIPS

Both the dichotomous scale (Table 4) and the Likert scale (Table 5) reveal significant findings worth mentioning. Firstly, according to the data registered at the end of the research, the group that participated in EOTIPS had fewer visits to the GP or neurologist due to stroke-related issues. This may be because they had more information regarding stroke care and potential difficulties at home (see Table 4). Secondly, opinions on public health-mediated rehabilitation and home care show higher satisfaction in the experimental group compared to the control group. Ultimately, most participants in the experimental group believed that EOTIPS had a positive impact on their recovery, with 82.1% rating it a 5 out of 5 and the remaining 17.9% rating it a 4 out of 5.

### 4.5. Strengths and Limitations

Within the limitations of the current study, we found that although all patients and caregivers of the experimental group received advice to continue rehabilitation at home following the occupational therapist’s guidance, the frequency and intensity of practice they performed at home depended on their willingness to train and the caregiver’s time availability. Other factors that could influence home training include the caregiver’s involvement and the patient’s adherence to treatment, which might also lead to bias in the results. Second, a semi-structured survey was completed at the end of the intervention. It was designed specifically for this research and has not been validated; therefore, the results should be considered cautiously. Nevertheless, this method allows researchers to delve deeper into specific areas of interest and gather rich, detailed data that may not emerge from more structured approaches. Third, the intervention was delivered by a single occupational therapist to ensure consistency across all sessions and to minimise variability in the therapeutic approach. However, this can introduce potential bias, as the therapist’s unique characteristics and approach may influence the results, significantly reducing the generalisability of the findings. Fourth, no data on caregiver characteristics were collected, which could have provided valuable insights for comparison and might have influenced the results. Published data suggest that stroke rehabilitation can be more successful if gender differences in caregivers’ adaptation to changes are considered. For example, men may require more counselling to adapt to the cognitive and emotional changes their spouses experience due to stroke [48]. Gender was not considered in the current analysis, but it could be an important factor for future research. It is also important to mention the potential impact of the dropout rate (2 deaths) and baseline differences between groups that could have affected the results, although no significant differences were found after the statistical analysis.

Regarding the strengths of the study, it demonstrates that involving caregivers in the patient’s recovery can potentially lead to better outcomes in caregiver burden and improve the patient’s quality of life, enhance their relationship, and promote rehabilitation at home. Additionally, there are few publications on interventions aimed at improving caregiver coping strategies for stroke patients, which can positively influence both caregivers’ experiences and patients’ recovery. Furthermore, the study showed a reduction in the frequency of visits to the GP or neurologist, which could imply a reduction in costs for the healthcare system and higher satisfaction for patients, although further research is needed to confirm this hypothesis.

## 5. Conclusions

The current study demonstrated a relationship between caregiver coping strategies, caregiver burden, and the quality of life of the patient. Therefore, by providing an intervention tailored to their needs and occupational balance, we can positively influence the caregiver’s burden and coping strategies and indirectly improve the patient’s quality of life. These findings may be useful for designing treatment interventions that support both the patient and the caregiver during the hospital-discharge process after a stroke. Further research could focus on long-term follow-up and exploring additional caregiver outcomes. It may also be of interest to investigate whether increasing the involvement of occupational therapy, in conjunction with interventions from other health professionals, would result in better outcomes for patients and their caregivers. Additionally, future studies should employ multiple therapists and incorporate measures to assess inter-rater reliability to further validate the findings.

## Figures and Tables

**Table 1:** Sociodemographic characteristics and clinical participants’ data in the EOTIPS study.

	Control (N = 30)	Experimental (N = 30)	*p*-Value
SEX			0.432 ^1^
Female	14 (46.66%)	11 (36.66%)	
Male	16 (53.33%)	19 (63.33%)	
AGE			0.479 ^2^
Mean (SD)	66.60 (10.60)	68.50 (10.70)	
EDUCATION			0.299 ^1^
No studies	1 (3.33%)	1 (3.33%)	
Knows how to read and write	7 (23.33%)	11 (36.66%)	
Primary School	15 (50.00%)	9 (30.00%)	
High School	5 (16.66%)	3 (10.00%)	
University	2 (6.66%)	6 (20.00%)	
MARITAL STATUS			0.974 ^1^
Single	2 (6.66%)	3 (10.00%)	
Divorced	2 (6.66%)	2 (6.66%)	
Widowed	5 (16.66%)	5 (16.66%)	
Married/living with partner	21 (70.00%)	20 (66.66%)	
NACIONALITY			0.278 ^1^
Spain	27 (90.00%)	24 (80.00%)	
Other countries	3 (10.00%)	6 (20.00%)	
NATIVE LANGUAGE			1.000 ^1^
Spanish	28 (93.33%)	28 (93.33%)	
Non-Spanish	2 (6.66%)	2 (6.66%)	
EMPLOYMENT STATUS			0.507 ^1^
Househusband/Housewife	1 (3.33%)	4 (13.33%)	
Unemployed	2 (6.66%)	1 (3.33%)	
Retired	19 (63.33%)	17 (56.66%)	
Temporary disability	8 (26.66%)	7 (23.33%)	
Other activities	0 (0.00%)	1. (3.33%)	
STROKE TYPE			0.052 ^3^
Ischemic	25 (83.33%)	30 (100.00%)	
Haemorrhagic	5 (16.66%)	0 (0.00%)	
DAMAGED HEMISPHERE			0.400 ^1^
Left	15 (50.00%)	19 (63.33%)	
Right	14 (46.66%)	11 (36.66%)	
Both hemispheres	1 (3.33%)	0 (0.00%)	
OXFORDSHIRE CLASSIFICATION			0.893 ^1^
LACS	16 (53.33%)	15 (50.00%)	
PACS	11 (36.66%)	10 (33.33%)	
POCS	2 (6.66%)	3 (10.00%)	
TACS	1 (3.33%)	2 (6.66%)	
SAQOL-39			0.029 ^3^
T1	2.51 (0.75)	2.67 (0.72)	
T2	3.41 (0. 69)	3.91 (0.70)	
BDI-II			0.011 ^3^
T1	13.60 (9.76)	11.23 (9.11)	
T2	15.30 (10.48)	8.40 (8.88)	
HAM-A			0.055 ^3^
T1	11.50 (10.88)	8.47 (10.96)	
T2	11.30 (9.00)	5.50 (7.94)	

^1^ Pearson’s Chi-squared test. ^2^ Student’s *t*-test. ^3^ Repeated measures ANOVA. Abbreviations: LACS = Lacunar syndrome; PACS = Partial Anterior Circulation Stroke; POCS = Posterior Circulation Stroke; TACS = Total Anterior Circulation Stroke; SAQOL-39 = Spanish Stroke and Aphasia Quality of Life Scale; BDI-II = Beck Depression Inventory and Aphasia Quality of Life Scale; T1 = initial evaluation; T2 = final evaluation: data presented as mean (SD).

**Table 2:** Patients–caregivers relationships.

	Control (N = 30)	Experimental (N = 30)	Total (N = 60)	*p*-Value
				0.756 ^1^
NO CAREGIVER	3 (5.00%)	2 (3.33%)	5 (8.33%)	
PARTNER	19 (31.66%)	18 (30.00%)	37 (61.66%)	
SONS	7 (11.66%)	6 (10.00%)	13 (21.66%)	
PARENTS	0 (0.00%)	1 (1.66%)	1 (1.66%)	
OTHERS	1 (1.66%)	2 (3.33%)	3 (5.00%)	
PRIVATE CAREGIVER	0 (0.00%)	1 (1.66%)	1 (1.66%)	

^1^ Pearson’s Chi-squared test.

**Table 3:** Results of repeated measures ANOVA for the outcome measures.

	Repeated Measures ANOVA								
			Control Group (*n* = 28) Mean (SD)	Experimental Group (*n* = 29) Mean (SD)	Time within-Subjects Factor	Group between-Subjects Factor	Treatment Time Interaction	POST HOC Exp vs. Cont and Size Effect	
CBS		T1	12.8 (12.6)	14.5 (6.91)	F = 1.68	F = 1.86	F = 6.04	T1	0.956 ^2^
		T2	3.91 (0.70)	3.41 (0.69)	df = 1.39	df = 1.39	df = 1.39	T2	0.199 ^2^
					*p* = 0.202 ^1^	*p* = 0.181 ^1^	*p* = 0.019 ^1^	*d* = 0.770 ^3^	
	Engagement strategies	T1	38.2 (12.6)	42.5 (10.2)	F = 11.60	F = 3.07	F = 2.26		
		T2	40.8 (13.6)	49.0 (10.5)	df = 1.36	df = 1.36	df = 1.36		
					*p* = 0.002 ^1^	*p* = 0.088 ^1^	*p* = 0.141 ^1^		
CSI	Problem-solving	T1	10.9 (2.65)	10.5 (2.56)	F = 0.238	F = 0.179	F = 0.113		
		T2	11.0 (2.57)	10.8 (2.61)	df = 1.36	df = 1.36	df = 1.36		
					*p* = 0.629 ^1^	*p* = 0.674 ^1^	*p* = 0.739 ^1^		
	Cognitive restructuring	T1	7.44 (5.14)	7.85 (4.48)	F = 2.46	F = 1.58	F = 3.76		
		T2	7.22 (4.41)	9.95 (2.72)	df = 1.36	df = 1.36	df = 1.36		
					*p* = 0.126 ^1^	*p* = 0.216 ^1^	*p* = 0.060 ^1^		
	Social support	T1	10.3 (5.04)	11.6 (4.19)	F = 22.18	F = 3.00	F = 4.68	T1	0.830 ^2^
		T2	11.7 (5.37)	15.3 (4.09)	df = 1.36	df = 1.36	df = 1.36	T2	0.103 ^2^
					*p* ≤ 0.001 ^1^	*p* = 0.092 ^1^	*p* = 0.037 ^1^	*d* = −0.703 ^3^	
	Express emotions	T1	9.56 (4.80)	12.6 (3.58)	F = 1.505	F = 4.75	F = 0.436		
		T2	10.9 (4.20)	12.9 (4.12)	df = 1.36	df = 1.36	df = 1.36		
					*p* = 0.228 ^1^	*p* = 0.036 ^1^	*p* = 0.513 ^1^		
	Disengagement strategies	T1	33.9 (6.80)	36.4 (8.18)	F = 3.661	F = 0.534	F = 0.472		
		T2	37.4 (7.95)	38.0 (7.60)	df = 1.36	df = 1.36	df = 1.36		
					*p* = 0.064 ^1^	*p* = 0.470 ^1^	*p* = 0.496 ^1^		
	Wishful thinking	T1	12.4 (3.40)	13.3 (3.39)	F = 0.112	F = 2.34	F = 1.666		
		T2	11.9 (4.07)	14.2 (3.44)	df = 1.36	df = 1.36	df = 1.36		
					*p* = 0.740 ^1^	*p* = 0.135 ^1^	*p* = 0.205 ^1^		
	Self-criticism	T1	5.11 (4.80)	7.00 (4.10)	F = 0.0207	F = 2.83	F = 0.2013		
		T2	4.72 (4.68)	7.20 (4.35)	df = 1.36	df = 1.36	df = 1.36		
					*p* = 0.886 ^1^	*p* = 0.101 ^1^	*p* = 0.656 ^1^		
	Problem avoidance	T1	5.83 (3.17)	6.30 (3.61)	F = 0.244	F = 0.0321	F = 0.842		
		T2	6.83 (4.19)	6.00 (4.23)	df = 1.36	df = 1.36	df = 1.36		
					*p* = 0.624 ^1^	*p* = 0.859 ^1^	*p* = 0.365 ^1^		
	Social withdrawal	T1	10.6 (2.66)	10.4 (2.96)	F = 9.73	F = 7.21	F = 13.88	T1	0.996 ^2^
		T2	14.0 (3.22)	10.1 (2.34)	df = 1.36	df = 1.36	df = 1.36	T2	<0.001 ^2^
					*p* = 0.004 ^1^	*p* = 0.011 ^1^	*p* ≤ 0.001 ^1^	*d* = −1.20 ^3^	

^1^ Repeated measures ANOVA. ^2^ Tukey test. ^3^ Cohen’s d effect. Abbreviations: CBS = Caregiver Burden Scale; CSI = Coping Strategies Inventory.

**Table 4:** Semi-structured survey: dichotomous scale.

	Control (N = 27)	Experimental (N = 28)	Total (N = 55)	*p*-Value
Do you think patient care has become easier over time?				0.121 ^1^
NO	11.0 (40.7%)	6.0 (21.4%)	17.0 (30.9%)	
YES	16.0 (59.3%)	22.0 (78.6%)	38.0 (69.1%)	
Has there been any readmission to the hospital since the stroke event?				0.970 ^1^
NO	11.0 (40.7%)	6.0 (21.4%)	17.0 (30.9%)	
YES	16.0 (59.3%)	22.0 (78.6%)	38.0 (69.1%)	
Have there been any consultations with the GP/neurologist for reasons related to the stroke?				0.004 ^1^
NO	7.0 (25.9%)	18.0 (64.3%)	25.0 (45.5%)	
YES	20.0 (74.1%)	10.0 (35.7%)	30.0 (54.5%)	
Are there other family members/friends involved in the patient’s care?				0.304 ^1^
NO	1.0 (3.7%)	0.0 (0.0%)	1.0 (1.8%)	
YES	26.0 (96.3%)	28.0 (100.0%)	54.0 (98.2%)	

^1^ Pearson’s Chi-squared test.

**Table 5:** Semi-structured survey: Likert scale.

	Control (N = 27)	Experimental (N = 28)	Total (N = 55)	*p*-Value
Do you feel satisfied with the recovery?				0.203 ^1^
1	2.0 (7.4%)	0.0 (0.0%)	2.0 (3.6%)	
2	4.0 (14.8%)	1.0 (3.6%)	5.0 (9.1%)	
3	2.0 (7.4%)	6.0 (21.4%)	8.0 (14.5%)	
4	11.0 (40.7%)	13.0 (46.4%)	24.0 (43.6%)	
5	8.0 (29.6%)	8.0 (28.6%)	16.0 (29.1%)	
Do you believe that the treatment provided in the current public healthcare is sufficient?				0.096 ^1^
1	2.0 (7.4%)	2.0 (7.1%)	4.0 (7.3%)	
2	4.0 (14.8%)	3.0 (10.7%)	7.0 (12.7%)	
3	5.0 (18.5%)	3.0 (10.7%)	8.0 (14.5%)	
4	5.0 (18.5%)	15.0 (53.6%)	20.0 (36.4%)	
5	11.0 (40.7%)	5.0 (17.9%)	16.0 (29.1%)	
Do you think that the current public health-mediated rehabilitation is enough?				0.011 ^1^
1	4.0 (14.8%)	1.0 (3.6%)	5.0 (9.1%)	
2	6.0 (22.2%)	3.0 (10.7%)	9.0 (16.4%)	
3	4.0 (14.8%)	5.0 (17.9%)	9.0 (16.4%)	
4	4.0 (14.8%)	16.0 (57.1%)	20.0 (36.4%)	
5	9.0 (33.3%)	3.0 (10.7%)	12.0 (21.8%)	
Do you think that the home care received by the public healthcare is sufficient?				<0.001 ^1^
1	17.0 (63.0%)	1.0 (3.6%)	18.0 (32.7%)	
2	5.0 (18.5%)	3.0 (10.7%)	8.0 (14.5%)	
3	3.0 (11.1%)	4.0 (14.3%)	7.0 (12.7%)	
4	1.0 (3.7%)	10.0 (35.7%)	11.0 (20.0%)	
5	1.0 (3.7%)	10.0 (35.7%)	11.0 (20.0%)	
Have you felt supported in the process by the assigned health professionals?				0.104 ^1^
1	3.0 (11.1%)	0.0 (0.0%)	3.0 (5.5%)	
2	0.0 (0.0%)	0.0 (0.0%)	0.0 (0.0%)	
3	5.0 (18.5%)	2.0 (7.1%)	7.0 (12.7%)	
4	5.0 (18.5%)	4.0 (14.3%)	9.0 (16.4%)	
5	14.0 (51.9%)	22.0 (78.6%)	36.0 (65.5%)	
Do you think that the information received about the stroke and its care was adequate at the time of discharge?				0.452 ^1^
1	4.0 (14.8%)	1.0 (3.6%)	5.0 (9.1%)	
2	4.0 (14.8%)	2.0 (7.1%)	6.0 (10.9%)	
3	7.0 (25.9%)	7.0 (25.0%)	14.0 (25.5%)	
4	5.0 (18.5%)	8.0 (28.6%)	13.0 (23.6%)	
5	7.0 (25.9%)	10.0 (35.7%)	17.0 (30.9%)	
In general, are you satisfied with the healthcare received?				0.400 ^1^
1	2.0 (7.4%)	1.0 (3.6%)	3.0 (5.5%)	
2	3.0 (11.1%)	0.0 (0.0%)	3.0 (5.5%)	
3	4.0 (14.8%)	4.0 (14.3%)	8.0 (14.5%)	
4	8.0 (29.6%)	9.0 (32.1%)	17.0 (30.9%)	
5	10.0 (37.0%)	14.0 (50.0%)	24.0 (43.6%)	
Do you think that the occupational therapy intervention received has had a positive effect on the recovery? (only answered by the experimental group)				
1		0.0 (0.0%)		
2		0.0 (0.0%)		
3		0.0 (0.0%)		
4		5.0 (17.9%)		
5		23.0 (82.1%)		

^1^ Pearson’s Chi-squared test.

## Data Availability

Trial registration: ClinicalTrials.gov NCT04835363. Registered on 25 March 2021.

## Supplementary Materials

The following supporting information can be downloaded at: https://www.mdpi.com/article/10.3390/jpm14080821/s1, Table S1: CONSORT 2010 checklist of information to include when reporting a randomised trial.
